# Method for Localization Aerial Target in AC Electric Field Based on Sensor Circular Array

**DOI:** 10.3390/s20061585

**Published:** 2020-03-12

**Authors:** Wenbin Zhang, Peng Li, Nianrong Zhou, Chunguang Suo, Weiren Chen, Yanyun Wang, Jiawen Zhao, Yincheng Li

**Affiliations:** 1 Faculty of Mechanical and Electrical Engineering, Kunming University of Science and Technology, Kunming 650504, China; lipeng@stu.kust.edu.cn (P.L.); cwrfjl@stu.kust.edu.cn (W.C.); 2 School of Electrical Engineering, Chongqing University, Chongqing 400044, China; zhounianrong@stu.kust.edu.cn; 3 Yunnan Power Grid Electric Power Research Institute of LLC, Kunming 650011, China; 4 Faculty of Science, Kunming University of Science and Technology, Kunming 650504, China; suochunguang@kust.edu.cn (C.S.); wangyanyun@stu.kust.edu.cn (Y.W.); zhaojiawen@stu.kust.edu.cn (J.Z.); liyincheng@stu.kust.edu.cn (Y.L.)

**Keywords:** safe distance, sensor circular array, target setting, layout optimization, localization error, voltage level

## Abstract

The traditional method of using electric field sensors to realize early warning of electric power safety distance cannot measure the distance of dangerous sources. Therefore, aiming at the electric field with a frequency of 50 to 60 Hz (AC electric field), a new method for localization of aerial AC target by the capacitive one-dimensional spherical electric field sensor circular array is studied. This method can directly calculate the distance, elevation, and azimuth of the detector from the dangerous source. By combining the measurement principle of the spherical electric field sensor and the plane circular array theory, a mathematical model for the localization of aerial targets in an AC electric field is established. An error model was established using Gaussian noise and the effects of different layout parameters on the localization error were simulated. Based on mutual interference between sensors, minimum induced charge, and localization error, an optimal model for sensor layout was established, and it was solved by using genetic algorithms. The optimization results show that when the number of sensors is 4, the array radius is 20 cm, and the sensor radius is 1.5 cm, the ranging error is 8.4%. The detector was developed based on the layout parameters obtained from the optimization results, and the localization method was experimentally verified at 10 and 35 kV alarm distances. The experimental results show that when the detector is located at 10 kV alarm distance, the distance error is 0.18 m, the elevation error is 6.8°, and the azimuth error is 4.57°, and when it is located at 35 kV alarm distance, the distance error is 0.2 m, the elevation error is 4.8°, and the azimuth error is 5.14°, which meets the safety distance warning requirements of 10 and 35 kV voltage levels.

## 1. Introduction

In order to ensure the safe and stable operation of a power system, it is necessary to constantly monitor and maintain live equipment and transmission lines. The monitoring methods currently used are: operator inspection [1], unmanned aerial vehicle (UAV) inspection [2], and electric inspection robot [3,4]. Regardless of the monitoring or maintenance of live equipment, people, UAV, and robots need to maintain a certain distance from high-voltage live bodies, which is called a safe distance [5]. If the safety distance is exceeded, the high-voltage environment will cause personal safety problems for the operator, and the complicated electromagnetic environment will cause interference with the control system of the UAV and robot, resulting in equipment failure [6]. Therefore, in order to ensure the personal safety of the operator and the reliability of the detection equipment, it is particularly important to accurately determine the safe distance from the live target and the location of the danger source. The common voltage levels and corresponding safety distances in the Chinese power industry are shown in Table 1.

Since the 20th century, electric field measurement technology has been greatly developed. The representative electric field measurement systems are Kapteos electric field measurement systems (Kapteos, Sainte-Hélène du Lac, France) and NBM-550 broadband field strength meter (Narda Safety Test Solutions GmbH, Pfullingen, Germany). Not only do they have high measurement accuracy, they can also be used in harsh environments. In recent years, the electric field measurement technology has gradually become smaller. The miniaturized sensor probe has the characteristics of portability, high measurement accuracy, and low cost [7,8,9,10]. The emergence of miniaturized electric field measurement devices has greatly promoted the development of electric power safety distance warning technology. At present, the technology of using electric field sensors for safe distance warning is used mainly to detect the electric field strength around a charged body through a portable electric field sensor, and uses a preset safety threshold to indirectly perform a safe distance warning [11,12,13]. However, the alarm thresholds at different safety distances are different for different voltage levels, so traditional methods are prone to false positives and false negatives. Reference [14,15] developed a DC electric field safety early warning device that can adapt to different voltage levels according to the voltage level identification algorithm. However, the algorithm of this method is difficult to implement and is not suitable for practical engineering problems. Therefore, there is a need for a safety early warning method that can directly obtain the distance of a dangerous target without being affected by the voltage level.

The technology of target localization and tracking has become a research hotspot in various fields [16,17,18,19]. Reference [20] proposed a WSN (wireless sensor network) positioning algorithm based on fuzzy decision-making. This algorithm can not only quickly locate the position of the nursing object in the hospital, but also has higher positioning accuracy than the traditional algorithm. Reference [21] used a scalar magnetometer orthogonal array to locate magnetic targets, and optimized the array structure by the Monte Carlo method, thereby improving the positioning performance. In reference [22], an acoustic locating method of array signal processing based on intersecting azimuth lines of two arrays is introduced. This method can locate moving sound sources with high accuracy (<5%). In reference [23], a planar triangle array was used to locate airborne electrostatic targets. This method determines the position of the target by the relationship between the time difference of the signal extreme value when the target flies through the array, the flying speed, and the strength of the target’s electrostatic field. This paper combines the planar circular array positioning technology and the electric field measurement technology. The proposed positioning method can directly obtain the coordinates of the danger source through the electric field sensor. The coordinates of the danger source can not only obtain the safety distance information, but can also calculate the area where the danger is located. In this way, operators and testing equipment can be safely exited from the danger zone.

The current research on electric field location technology is based on the electrostatic field or high-frequency partial discharge signals [24,25] and the power–frequency electric field is the most widely used in the power industry. Power–frequency electric field refers to the electric field with a frequency of 50 to 60 Hz. It belongs to a quasi-static field and can also be analyzed by the theory of electrostatic fields [26]. Therefore, the electrostatic positioning theory can be applied to the positioning of AC charged targets. Literature [27] studied the characteristics of airborne electrostatic targets and proposed that electrostatic targets are point charges when the measurement distance is greater than 3 to 6 times the target size. Reference [28] proposed that when the distance between the target and the sensor is 10 times the radius of the sensor plate, the target is a point charge and the electric field generated is a uniform electric field. They provided a theoretical basis for the equivalent of a charged body as a point target in the research of the power industry.

According to the analysis of the research status, a method of air target location for an AC electric field is proposed for drones, electric operators and inspection robots. The coordinates of the target can be calculated by measuring the amount of induced charge of each sensor in the circular array. An error model was established by analyzing the cause of the error, and the optimal layout parameters were calculated using a genetic algorithm based on the error model. The detector was designed based on the optimized results and experimentally verified in the laboratory. The experimental results show that the mathematical model can determine the location of the dangerous source, and it can be used for 10 and 35 kV safety warning in the power industry within the tolerance range.

This article is divided into seven parts: Section 1 is the introduction; Section 2 is the localization principle; Section 3 is the error analysis; Section 4 is the establishment of the sensor layout optimization model; Section 5 is the solution of the optimization model; Section 6 is the experimental design and Section 7 is the conclusion.

## 2. Localization Principle

**Figure 1:**
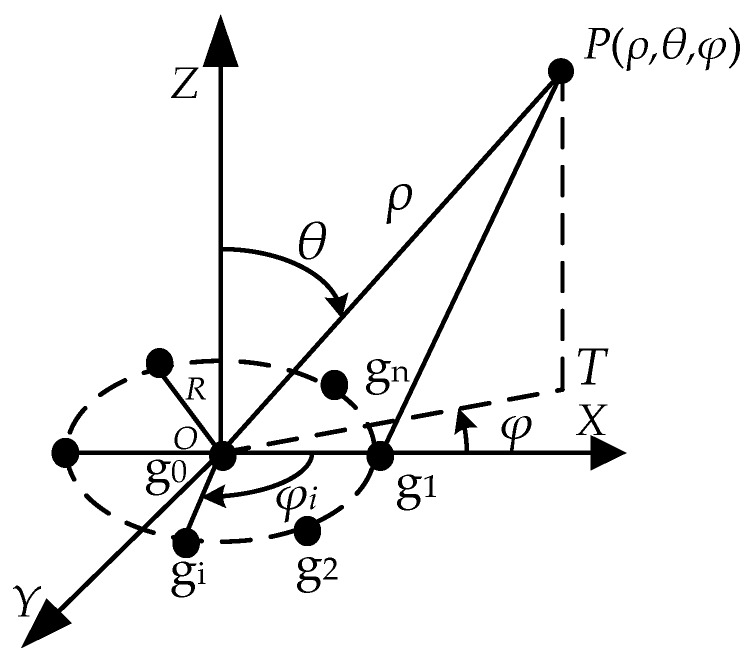
Schematic diagram of the detector.

Figure 1 is a schematic diagram of the detector. We set the spherical coordinate system with the center *O* of the array as the origin. A sensor g_0_ with a radius *r* is arranged in the center of the array, and the remaining *n* sensors g_1_, g_2_, …, g_n_ are evenly arranged on the circumference. *R* is the radius of the array, and the charge amount of the air-charged target *P* in the air is Q. The distance from the geometric center of the target to the center of the array is *ρ*, the azimuth of the target *φ* is the angle between the projection *OT* of *ρ* on the *OXY* plane and the *X* axis, the elevation angle *θ* of the target is the angle between the *Z* axis and *ρ*, and the angle between the *i* sensor and the *X* axis is *φ_i_*.

(Note: The sensors mentioned before in Section 6 of this article are all capacitive spherical AC electric field sensors).

The relationship between the electric field strength $E_{i}$ at a point in space and the charge Q of the target can be expressed as [29]

(1) $$E_{i}=\frac{Q}{4\pi \varepsilon_{0}\rho_{i}^{2}},$$

where $\varepsilon_{0}$ is the dielectric constant of the vacuum and $\rho_{i}$ is the distance between the spherical sensor on the circumference and the geometric center of the target.

According to the Gauss theorem and the measuring principle of the spherical electric field sensor, the sensor’s induced charge $Q_{i}$ can be expressed as [30]

(2) $$Q_{i}=\int \sigma dS\approx 3\pi \varepsilon_{r}r^{2}E_{i},$$

where σ is the charge density, S is the effective area of the sensor, $\varepsilon_{r}$ is the dielectric constant of the medium between the plates, and r is the radius of the sensor.

By introducing Equation (2) into Equation (1), the relationship between the sensor’s induced charge amount $Q_{i}$ and the target charge amount can be expressed as

(3) $$Q_{i}=\frac{3\varepsilon_{r}r^{2}}{4\varepsilon_{0}}\frac{Q}{\rho_{i}^{2}}=K\frac{Q}{\rho_{i}^{2}},$$

where $K=(3\varepsilon_{r}r^{2})/(4\varepsilon_{0})$ makes Equation (3) easy to write.

Combining the geometric position relationship shown in Figure 1 and the circular array localization theory, we can get

(4) $$\frac{KQ}{Q_{i}}=\frac{KQ}{Q_{0}}+R^{2}-2R\sqrt{\frac{KQ}{Q_{0}}}\sin\theta \cos(\varphi -\varphi_{i}),$$

where $Q_{0}$ is the induced charge of the intermediate sensor of the array. Sum n equations in Equation (4), and we can get

(5) $$\sum_{i=1}^{n}\frac{KQ}{Q_{i}}=n\frac{KQ}{Q_{0}}+nR^{2}-2R\sqrt{\frac{KQ}{Q_{0}}}\sin\theta \sum_{i=1}^{n}\cos(\varphi -\varphi_{i}).$$

Since the number of sensors n on the circumference is even and the sensors are symmetrically distributed, we can get

(6) $$\left\{ \begin{array}{l} \sum_{i=1}^{n}\sin m(\varphi -\varphi_{i})=0\\ \sum_{i=1}^{n}\cos(m\varphi -m\varphi_{i})=0 \end{array}\right.\left(m=1,2,3\right).$$

Bring Equation (6) into Equation (5) to get the distance from the center of the array to the target $\rho_{0}$ and express it as

(7) $$\rho_{0}=\frac{R\sqrt{n}}{\sqrt{\sum_{i=1}^{n}\left(\frac{Q_{0}}{Q_{i}}-1\right)}}.$$

The elevation equation and azimuth equation of the target can be obtained by using the least squares algorithm. Let y be the cumulative sum of the squared errors of the equations in Equation (5); we can get

(8) $$y=\sum_{1}^{n}e_{i}^{2},$$

where $e_{i}$ is the error of each equation, in Equation (8), and express it as

(9) $$\begin{array}{l} e_{i}=\sum_{i=1}^{n}\frac{KQ}{Q_{i}}-n\frac{KQ}{Q_{0}}-nR^{2}+2R\sqrt{\frac{KQ}{Q_{0}}}\sin\theta \sum_{i=1}^{n}\cos(\varphi -\varphi_{i})\\ \left(i=1,2,\ldots ,n\right) \end{array}$$

Substituting Equation (9) into Equation (8), and let ∂y/∂θ=0,∂y/∂φ=0, we can get

(10) $$\left\{ \begin{array}{l} \sum_{1}^{n}\frac{KQ}{Q_{i}}\cos(\varphi -\varphi_{i})-\frac{KQ}{Q_{0}}\sum_{1}^{n}\cos(\varphi -\varphi_{i})-R^{2}\sum_{1}^{n}\cos(\varphi -\varphi_{i})+2R\sqrt{\frac{KQ}{Q_{0}}}\sin\theta \sum_{1}^{n}\cos^{2}(\varphi -\varphi_{i})=0\\ \sum_{1}^{n}\frac{KQ}{Q_{i}}\sin(\varphi -\varphi_{i})-\frac{KQ}{Q_{0}}\sum_{1}^{n}\sin(\varphi -\varphi_{i})-R^{2}\sum_{1}^{n}\cos(\varphi -\varphi_{i})+2R\sqrt{\frac{KQ}{Q_{0}}}\sin\theta \sum_{1}^{n}\sin(2\varphi -2\varphi_{i})=0 \end{array}\right.$$

Using Equation (6) to simplify Equation (10), we can get

(11) $$\sin\theta =-\frac{Q_{0}\rho_{0}}{nR}\sum_{1}^{n}\frac{\cos\varphi \cos\varphi_{i}+\sin\varphi \sin\varphi_{i}}{Q_{i}}.$$

Using the relation of trigonometric functions, Equation (11) can be transformed into

(12) $$\sin\theta =\frac{Q_{0}\sqrt{{\left(\sum_{1}^{n}\frac{\cos\varphi_{i}}{Q_{i}}\right)}^{2}+{\left(\sum_{1}^{n}\frac{\sin\varphi_{i}}{Q_{i}}\right)}^{2}}}{\sqrt{n\sum_{1}^{n}\left(\frac{Q_{0}}{Q_{i}}-1\right)}}.$$

From Equation (10), we can get (Equation (13))

(13) $$\sum_{1}^{n}\frac{\sin\varphi \cos\varphi_{i}}{Q_{i}}=\sum_{1}^{n}\frac{\cos\varphi \sin\varphi_{i}}{Q_{i}}.$$

Use the relationship of trigonometric functions, we can get

(14) $$\tan\varphi =\frac{\sum_{1}^{n}\frac{\sin\varphi_{i}}{Q_{i}}}{\sum_{1}^{n}\frac{\cos\varphi_{i}}{Q_{i}}}.$$

Using Equations (7), (12) and (14), the distance, elevation, and azimuth of the field source can be obtained.

The localization method in this paper is not affected by the voltage level. From Equation (15), the target voltage level U is directly proportional to the amount of charge Q it carries and inversely proportional to the capacitance C. Capacitance is only related to the structure of the target, so for targets of the same structure, the target voltage level is only related to the amount of charge. According to Equation (3), it can be seen that as the target charge increases by ζ times, the amount of induced charges on the surface of each sensor also increases by ζ times, and ζ will be reduced to a fraction during the calculation of the localization equation.

(15) $$U=\zeta \frac{Q}{C}.$$

## 3. Error Analysis

### 3.1. Error Model

AC electric field localization errors are mainly composed of errors caused by sensor layout and errors during electric field measurement. It can be seen from $\rho_{0}=\frac{R\sqrt{n}}{\sqrt{\sum_{i=1}^{n}\left(\frac{Q_{0}}{Q_{i}}-1\right)}}$ that the target distance is related to the amount of sensor charge, the radius of the detector, and the number of sensors (called the layout parameters). In the actual measurement process, the sensor layout parameters determine the distance between the sensors. If the distance between the sensors is too small, it will cause coupling interference between the sensors. The noise in the AC signal received by the sensor will also affect the measurement accuracy. During the measurement, it will be filtered, but it still cannot completely eliminate the impact of the noise of other frequency components in the AC signal and the random noise of the circuit itself.

According to a large number of measurement practices and research by Chen Xi of Beijing Institute of Technology [31,32], most of the noise in the measurement process is Gaussian white noise, and the noise $Q_{{}_{error}}^{i}$ generated during the measurement process can be expressed as

(16) $$Q_{{}_{error}}^{i}=normrnd\times Q_{i}\times k_{e},$$

where normrnd is a random number obeying the standard normal distribution, and $k_{e}$ is the noise weight, and its size is related to the environment and the measurement circuit. The $k_{e}$ in this paper is 0.01 [33]. Through the error analysis, the distance error caused by noise can be expressed as

(17) $$\sigma_{p}=\left|\frac{\rho_{0}-\rho_{0}^{\prime }}{\rho_{0}}\right|,$$

where $\sigma_{p}$ is the distance error, which represents the ratio of the distance error value to the actual value, and $\rho_{0}^{\prime }$ is the distance error after adding noise, which can be expressed as

(18) $$\rho_{0}^{\prime }=\frac{R\sqrt{n}}{\sqrt{\sum_{i=1}^{n}\left(\frac{Q_{0}^{\prime }}{Q_{{}_{i}}^{\prime }}-1\right)}},$$

where $Q_{0}^{\prime }$ and $Q_{i}^{\prime }$ are the simulated charge amounts of the electric field sensor at the center of the array and the electric field sensor at the circumference, respectively. The simulated charge is the superposition of the theoretical calculated value and the error value, which is expressed as:

(19) $$Q_{i}^{\prime }=Q_{i}+Q_{{}_{error}}^{i}.$$

The simulation charge amount of each sensor can be obtained through Equation (19), and then the distance measurement accuracy can be calculated from Equation (17). The calculation of elevation and azimuth errors is the same.

### 3.2. Influence of Layout Parameters on Measurement Errors

The layout parameters that affect the measurement error of the AC electric field detector are: number of sensors, array radius, and sensor size. Increasing the radius of the detector and the radius of the sensor will cause the amount of the sensor’s induced charge to change. From Section 2, it can be seen that noise is related to the amount of induced charge. Noise is the key factor that affects the measurement error, so the influence of different array radii and sensor radii on the measurement error needs to be studied. It can also be seen from the localization equation that the measurement error is related to the number of sensors.

In order to study the influence of the array radius on the measurement error, a Monte Carlo method was used for simulation. After setting the simulation conditions, the computer generates random numbers that obey the standard normal distribution, and the error caused by noise is combined with the theoretical induced charge through Equation (16) and Equation (19) to obtain the simulation value. Finally, the simulation value is taken into Equation (18) for calculation, and the influence of different layout parameters on the distance error is obtained. Elevation error and azimuth error are the same.

#### 3.2.1. Value Range of Layout Parameters

The localization system studied in this article will be applied to UAV, human bodies, and robots, so the detector volume should not be too large. According to the size of most UAV wings and helmets in the market, the range of the array radius should be 0.09 to 1 m.

If the radius of the sensor is too small, the detection distance of the detection system cannot meet the requirements of the safe distance warning, and if the radius of the sensor is too large, the distance between the sensors will be reduced and the measurement accuracy will be affected [31]. After our experiments, we found that when the voltage of the field source is 110 kV, the measurement distance of the flat-type electric field sensor with a radius of 6 cm can reach at least 10 m. When the voltage of the field source is 10 kV, the flat-type electric field sensor with a radius of 1 cm can measure a distance of at least 3 m. Therefore, the radius of the sensor in this paper is 1 to 6 cm.

Too many sensors will not only cause the sensor pitch to be too close, but will also bring higher costs. When the minimum radius of the sensor is 1 cm, the maximum radius of the array is 1 m, the number of sensors on the circumference is 30, and the ratio of the sensor radius to the sensor spacing is less than 0.05: the coupling interference between the sensors can be reduced (see Section 4.1 for an explanation). In order for Equation (6) to be true, the number of sensors *n* on the circumference must be even, and in order to locate the target in the air, the number of sensors *n* on the circumference must be greater than or equal to 4, so the range of the number of sensors is *n* + 1 = [5,31].

#### 3.2.2. Simulation Analysis of the Effect of the Number of Sensors on the Localization Error

In order to obtain the influence of the number of different sensors on the localization error, the simulation method in the previous section was used for research. The simulation conditions are set as follows: the target is a point charge with alternating current, and its charge is 10^−6^ C. The center of the detector is 10 m away from the target, the elevation angle is 30°, the azimuth angle 30°, the radius of the array is 0.1 m, the radius of the sensor is 1 cm, and the range of the number of sensors is *n* + 1 = [5,31].

**Figure 2:**
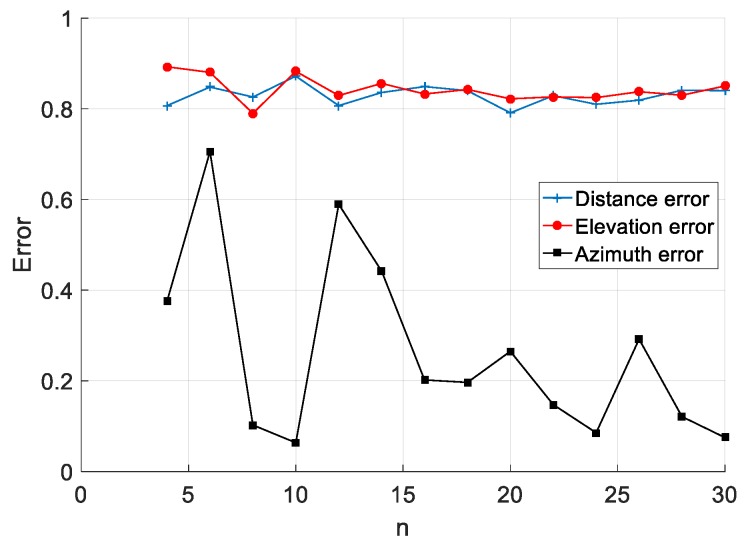
Relationship between number of sensors and localization error.

The simulation results are shown in Figure 2, where the abscissa is the number of sensors *n* on the circumference, and the ordinate is the localization error, which includes: distance error, elevation error, and azimuth error, which are all real numbers. It can be seen from the simulation results that with the increase of *n*, the value of the distance error and elevation angle error has a small effect, but the effect on the azimuth error has a large effect. Among the results, the distance error only changed from 0.791 to 0.849 and they differed by 0.058, the elevation error changed from 0.790 to 0.8927 and they differed by 0.1027, and the azimuth error changed from 0.0635 to 0.7055 and they differed by 0.642.

#### 3.2.3. Simulation Analysis of the Influence of Array Radius on Localization Error

The main purpose of this article is to measure the distance of the target. It can be seen from Figure 2 that the distance error is the smallest when the number of sensors is 20. Therefore, in this section, the number of sensors is 20, and the range of the array radius is 0.09 to 1 m. Other simulation conditions are the same as those in the previous section. 

**Figure 3:**
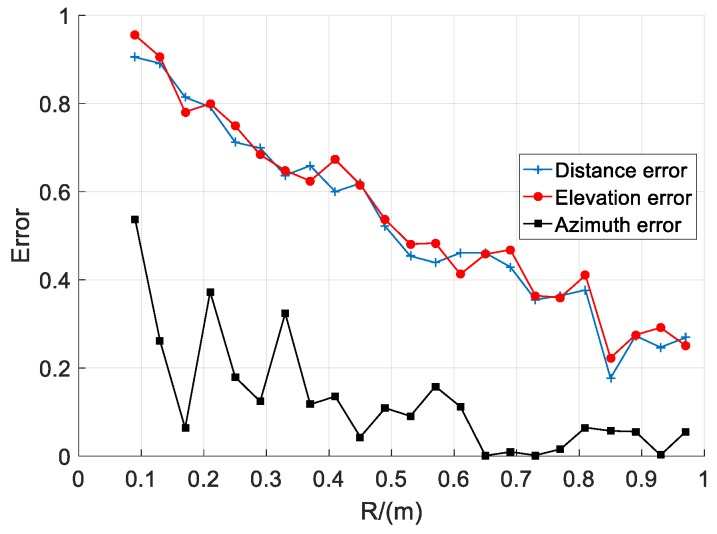
Relationship between array radius and localization error.

The simulation results are shown in Figure 3, where the abscissa is the array radius *R* and the ordinate is the localization error. From the simulation results, it can be seen that with the increase of *R*, the values of the distance error, elevation error, and azimuth error have a greater impact. Among them, the distance error changed from 0.9050 to 0.1770 and they differed by 0.728, the elevation error changed from 0.9547 to 0.2230 and they differed by 0.7317, and the azimuth error changed from 0.5377 to 0.0020 and they differed by 0.5177. It can also be seen from Figure 3 that the localization error decreases as the array radius increases.

#### 3.2.4. Simulation Analysis of the Effect of Sensor Radius on Localization Error

It can be seen from Figure 3 that the distance error is the smallest when the array radius is 0.84 m. Therefore, in this section, the array radius is taken as 0.84 m, and the range of the sensor radius is taken as 1 to 6 cm. The rest of the simulation conditions are the same as in Section 3.2.1.

**Figure 4:**
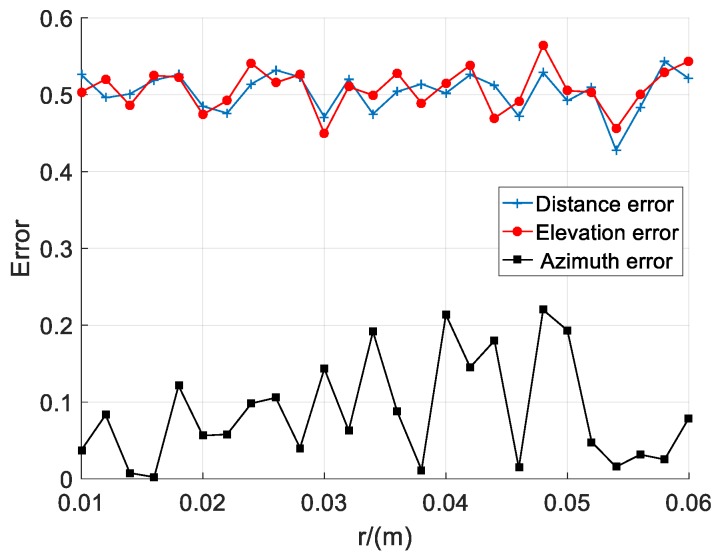
Relationship between sensor radius and localization error.

The simulation results are shown in Figure 4, where the abscissa is the sensor radius *r* and the ordinate is the localization error. From the simulation results, we can see the effect of *r* on distance error, elevation error and azimuth error. The distance error changed from 0.428 to 0.543 and they differed by 0.115, the elevation error changed from 0.4493 to 0.5643 and they differed by 0.115, and the azimuth error changed from 0.0023 to 0.22 and they differed by 0.2177.

It can be seen from the simulation results of Figure 2, Figure 3 and Figure 4 that different layout parameters have different degrees of influence on the distance error. The influence of the array radius on the distance error is large, while the number of sensors and the radius of the sensor have a small effect on the distance error.

## 4. Building an Optimization Model

### 4.1. Determination of Constraints

Human, animal, and sensor induction plates are conductors. When the conductors are in an electric field, they also generate an electric field, which is superimposed with the original electric field to form a distortion electric field [34]. In the actual measurement process, the sensor will not only distort the electric field at its location, but also affect the measurement results of the surrounding sensors. As a result, a large electric field measurement error is caused. Increasing the sensor spacing can reduce this error, so how much sensor spacing needs to be studied to reduce this effect. Reference [30] found that the mutual interference between sensors can be ignored when the ratio between the distance between two sensors and the radius of the sensor is less than or equal to 0.05 by the mirror method. Therefore, the first constraint is expressed as:

(20) $$r-R\sin(\frac{\pi }{n})/10\leq 0.$$

The signal obtained by the sensor comes from the amount of charge induced on the surface. When the amount of induced charge is accumulated to a certain amount, the matching circuit at the front end can respond, which is called the minimum amount of induced charge. The sensor will not be able to detect the correct signal if it is less than the minimum induced charge amount, so the sensor’s induced charge amount should be greater than the minimum induced charge amount $Q_{\min}$, and combining $Q_{i}=\frac{3\varepsilon_{r}r^{2}}{4\varepsilon_{0}}\frac{Q}{\rho_{i}^{2}}=K\frac{Q}{\rho_{i}^{2}}$, the second constraint can be expressed as

(21) $$Q_{\min}-\frac{3Q\varepsilon_{r}}{4\varepsilon_{0}}\frac{r^{2}}{\rho_{i}^{2}}\leq 0.$$

**Figure 5:**
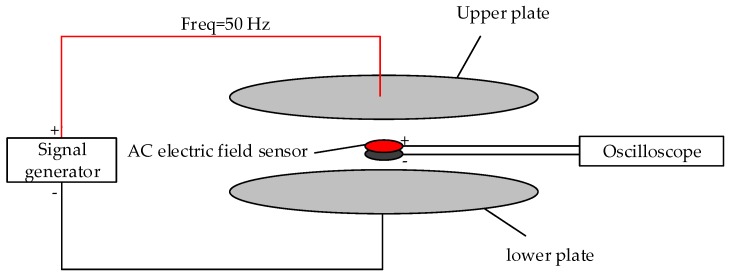
Block diagram of the experimental platform.

Among them, in order to have a feasible solution to the objective function, the value of the minimum induced charge amount needs to be known in advance. For this reason, the following experiments are designed in this paper. The AC voltage with a frequency of Freq = 50 Hz is generated by the signal generator and applied to the upper plates of the calibration device with a distance of d = 3.2. The lower plate is connected to the low of the signal generator. In this way, a uniform 50 Hz electric field with controllable amplitude can be generated. Then, the positive and negative ends of the capacitive electric field sensor without a conditioning circuit are connected through an oscilloscope and the waveform is displayed. Finally, observe the change of the oscilloscope waveform to get the minimum induced charge of the sensor. The block diagram of the experimental platform is shown in Figure 5.

According to the analysis in Section 3.2, the layout parameters have a great influence on the distance error, and the maximum can reach 0.728 (72.8%). In the safety distance warning process, the warning is usually started at 1.5 times the safety distance. Taking a charged target of 110 kV as an example, its safety distance is 1.5 m, so the warning device should alarm at 2.25 m, so the alarm error must not be greater than 0.75 m. In order to better protect personal safety, the measurement accuracy should be limited to a higher range, so the distance error is controlled within 18%. Therefore, the third constraint is expressed as

(22) $$\sigma_{p}-0.18\leq 0.$$

**Figure 6:**
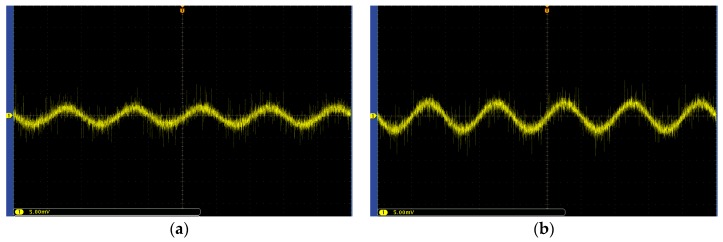
Waveform change diagram: (**a**) electric field strength is 0–31.3 V/m; (**b**) the electric field strength is 62.5 V/m.

The test results are shown in Figure 6, where the abscissa is time and the ordinate is voltage amplitude. Figure 6a shows the waveform when the field strength is 0 to 31.3 V/m. During the process of gradually increasing the voltage, it is found that the waveform remains basically unchanged in this field strength range. Figure 6b shows the waveform when the field strength is directly increased from 31.3 to 62.5 V/m. It can be seen that the amplitude of the waveform has been significantly enhanced. The experimental results show that the sensor can respond when the applied uniform field strength is 62.5 V/m. Therefore, the induced charge calculated based on $Q_{i}=\int \sigma dS\approx 3\pi \varepsilon_{r}r^{2}E_{i}$ of 1.3 × 10^−12^ C can be used as the minimum induced charge of the sensor designed in this paper. The sensor is a circular plate capacitive sensor with a radius of 1 cm.

When the distance between the sensors is too small, the measured values between the sensors are too close, and the difference between them cannot be distinguished, so it cannot be used for localization. Therefore, the amount of induced charge between the two sensors should be greater than or equal to $Q_{\min}$. Therefore the fourth constraint is expressed as

(23) $$Q_{\min}-(\left|Q_{i+1}-Q_{i}\right|,\left|Q_{i}-Q_{i-1}\right|)\leq 0.$$

### 4.2. Determination of the Objective Function

According to the reference [35], it can be known that using as many sensors as possible can increase the localization accuracy, and the larger the sensor radius in the measurement process, the greater the amount of received charges and the higher the system resolution. The simulation results in Section 3.2.3 can tell us that the larger the array radius, the smaller the measurement error. So our goal is to use layout parameters that have more sensors, larger sensor radius, and larger array radius, but considering that some conditions of the actual application scenario will contradict the above analysis, this can restrict the parameters to a certain range. It can be seen that this problem is a multi-objective optimization problem, so an evaluation function needs to be established to convert the multi-objective optimization problem into a single-objective optimization solution. The main purpose of this article is the measurement of distance, so the evaluation function is set as follows

(24) $$F=(\sigma ,R,r,n)=k_{\sigma }\sigma +k_{R}\frac{1}{R}+k_{r}\frac{1}{r}+k_{n}\frac{1}{n},$$

where $k_{\sigma }$, $k_{R}$, $k_{r}$, $k_{n}$ are the weights of each objective function in the evaluation function. Their sum is 1, which can be expressed as

(25) $$k_{\sigma }+k_{R}+k_{r}+k_{n}=1.$$

Through the above analysis the optimization model can be expressed as

(26) $$\begin{array}{l} Objective\;function:J=\min\left\{F(\sigma_{P},R,r,n)\right\}\\ Constraint:s.t\left\{ \begin{array}{l} r-R\sin(\frac{\pi }{n})/10\leq 0\\ Q_{\min}-\frac{3Q\varepsilon_{r}r_{i}{}^{2}}{4\varepsilon_{0}\rho_{i}{}^{2}}\leq 0\\ \sigma_{P}-0.18\leq 0\\ Q_{\min}-(\left|Q_{i+1}-Q_{i}\right|,\left|Q_{i}-Q_{i-1}\right|)\leq 0 \end{array}\right. \end{array}$$

## 5. Optimization of Sensor Layout Parameters Based on Genetic Algorithm

### 5.1. The Solution Process of Genetic Algorithm

The fitness function must be established before solving the model. The advantage of the genetic algorithm is that it is possible to distinguish the good or bad individuals in the population only by using the fitness function without resorting to external information. The higher the fitness, the higher the probability of being selected is closer to the optimal solution. The fitness function is usually the objective function, but the genetic algorithm used in this article is an optimization algorithm to solve the minimum value, so the fitness function is expressed as

(27) $$f(\sigma_{p},R,r,n)=\frac{1}{J}.$$

**Figure 7:**
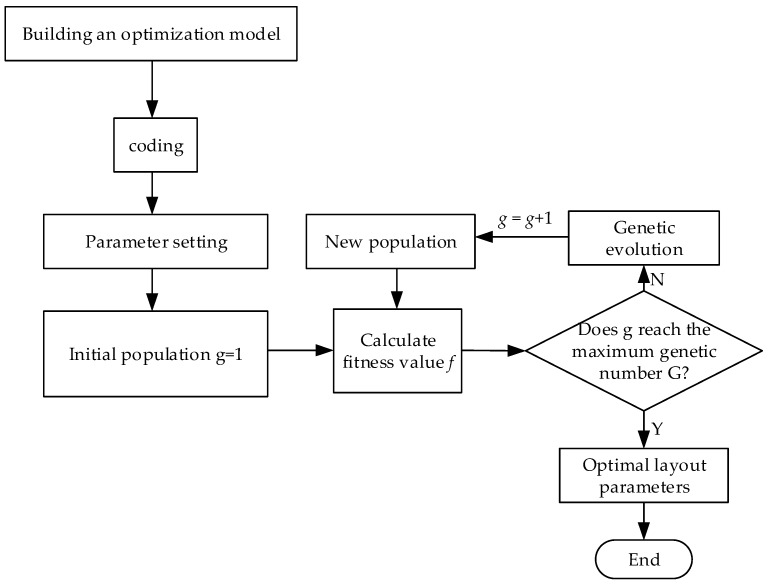
Algorithm solving flowchart.

The algorithm solving process is shown in Figure 7. The specific steps are as follows:

**Step** **1:** Establish an optimization model based on the localization principle and error analysis.
**Step** **2:** Determine the encoding method. The mathematical equation of the objective function and constraints in this article is a function optimization problem, so the real number encoding is selected.
**Step** **3:** Configure the optimization model and genetic algorithm related parameters according to Table 2 and Table 3.
**Step** **4:** Initialize the population. The optimization of the layout parameters in this article belongs to a more complicated optimization problem. Therefore, the number of the initial population is set to 200, and the current generation number is g = 1.
**Step** **5:** According to Equation (27), calculate the fitness value of each individual in the contemporary population, and rank the fitness from large to small.
**Step** **6:** Judgment of termination condition. If g ≥ G = 500, the operation is terminated, and the most adaptive individual in the current population is the optimal solution; if g < G, the operation continues, and step 7 is performed.
**Step** **7:** Genetic evolution operation. Genetic evolution includes selection, crossing, and mutation. It is the most basic component of genetic algorithm [36]. After the operation is completed, a new generation of population will be generated. At this time, *g* = *g* + 1, go to Step 5.

### 5.2. Simulation Experiments and Optimization Results

Before using the genetic algorithm to solve, we need to set the relevant parameters, which are related to the use background. First, according to the degree of influence of different layout parameters on measurement errors in Section 3, the weights of each objective function can be determined. Secondly, this article’s application is mainly installed and worn on UAV, power workers (hand-held), and substations. Therefore, the value range of some parameters needs to be limited to meet engineering needs. The parameter settings of the optimization model are shown in Table 2, and the genetic algorithm parameter settings are shown in Table 3.

Table 4 shows the results of the optimization. It is known from the results that when the number of sensors is 4, the evaluation function obtained is less than 6. This is because due to the existence of errors, too many sensors will cause error accumulation to make the distance error $\sigma_{p}$ larger, and the weight $\sigma_{p}$ of $k_{\sigma }$ in the evaluation function has the highest proportion. Therefore, this paper selects the layout parameter group with 4 sensors arranged on the circumference, and then selects the layout parameter with the evaluation function of 8.65588 in Table 3 according to the minimum principle. When using this set of layout parameters, the distance error is 8.4%.

## 6. Experimental Verification

### 6.1. Detector Hardware Design

**Figure 8:**
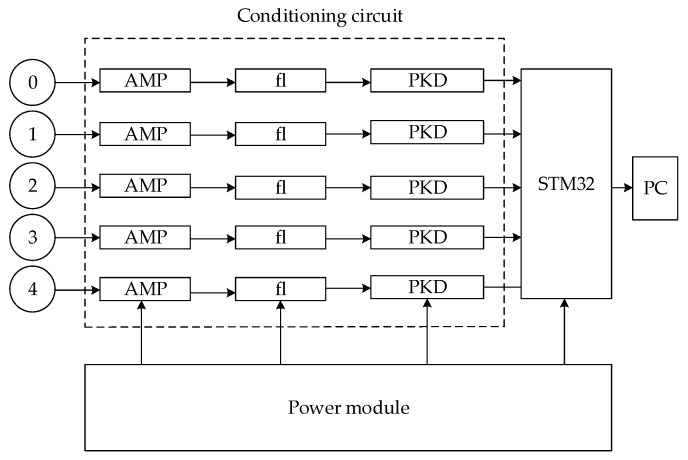
Detector circuit block diagram.

The circuit block diagram of the detector is shown in Figure 8. The induction plate of the electric field sensor will generate an induced charge that changes with the frequency of the electric field in the electric field. The induced charge is converted into a voltage signal by $U=\zeta \frac{Q}{C}$ for measurement. At this time, the voltage signal collected by the sensor is very weak, usually millivolt level, so it needs to be amplified by the amplifier circuit in the conditioning circuit. The amplified signal has not only the 50 Hz power–frequency signal, but also the noise of other components, so it is necessary to filter the signals of other components through a filter circuit (fl). The rectified signal is a standard sine wave. The filtered signal is converted into a DC signal by a peak detection circuit (PKD). The DC signal is transmitted to the STM32 processor for subsequent operations. 

The sensors used in this section perform linear calibration of the voltage amplitude and uniform field strength of the sensor output in a uniform electric field environment, so that the voltage amplitude of the sensor output can represent the field strength value. (Note: The sensors mentioned in Section 6 are one-dimensional circular capacitive power–frequency electric field sensors).

**Figure 9:**
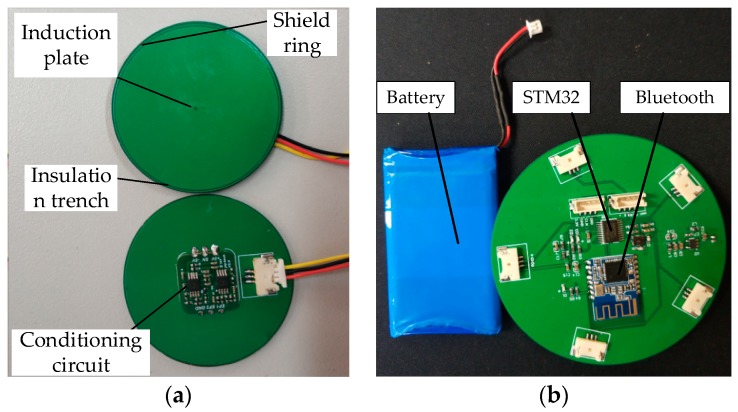
Measuring device: (**a**) electric field sensor; (**b**) controller.

The ball sensor is difficult to process, costly and not easy to carry, while the measurement accuracy of the flat-type electric field sensor is close to that of the spherical sensor [37,38]. Therefore, the sensor used in this paper is a circular capacitive power–frequency electric field sensor. Based on the optimized layout parameter group, a flat-type power–frequency electric field sensor as shown in Figure 9a was developed. The front of the sensor was composed of an induction plate and a shielding ring, which were separated by an insulation trench. The shield ring is connected to the lower plate and is connected to the grounding end of the circuit. It can shield part of the electric field radiated from the edge of the induction plate, and also shield part of the electric field radiated from the surrounding electric field sensor, thereby improving the measurement accuracy. The radius of the induction plate is 1.5 cm, the width of the shielding ring is 0.5 mm, and the width of the insulation trench is 0.5 mm. The back of the sensor is composed of a lower-level board and a conditioning circuit. To avoid the weak analog signal from the sensor being affected by the electric field before it reaches the conditioning circuit, the conditioning circuit is directly attached to the lower-level board of the sensor.

The acquisition system is shown in Figure 9b, which can simultaneously collect analog signals output by five sensors, and convert the analog signals into digital signals through the A/D module. The coordinates of the target are calculated by the STM32 controller. Finally, the output value of each sensor and the coordinates of the target are sent to the PC for display via the Bluetooth module. The power supply mode is powered by a 5 V lithium battery.

### 6.2. Experimental Platform Construction and Experimental Analysis

**Figure 10:**
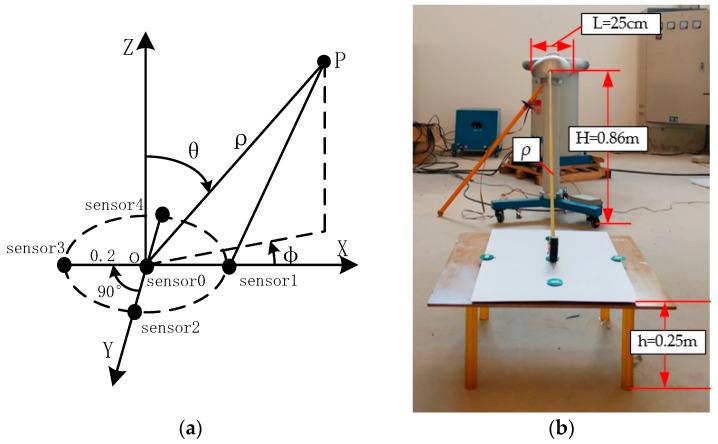
Experiment: (**a**) optimized detector schematic; (**b**) experimental device.

Based on the optimized results, a schematic diagram of the detector in Figure 10a is designed. A spherical coordinate system is established by using the detector’s original center *O*. The one-dimensional circular capacitive power–frequency electric field sensors with a radius of 1.5 cm and a thickness of 1.5 mm of the four induction plates are uniformly arrayed on the same circumference, and a sensor is also arranged in the middle; the array radius is 20 cm. In order to verify the positioning method of the AC electric field target proposed in this paper, the test platform of Figure 10b was designed. The AC high-voltage test bench with a height of H = 0.86 m from the ground can generate a stable voltage of 50 Hz, and its maximum size is L = 25 cm. Literature [27] mentioned that when the distance between the target and the detector is more than 3 to 6 times the size of the target, the electrostatic target is a point charge. Therefore, in order to meet the conditions of the point target, the measurement distance *ρ* should be ≥ 0.75 m.

In order to prevent the high-voltage from causing life safety problems for the experimenters, the measurement is only performed in a 5 kV voltage environment in this experiment. Localization data for other voltage levels can be obtained by changing the ζ value of $U=\zeta \frac{Q}{C}$.

The measurement methods and experimental steps of each localization parameter are introduced below.

1. Elevation Measurement

Place the detector on an insulating table with a height of *h*_1_ = 0.25 m above the ground and a carton with a height of *h*_2_ = 0.15 m from the ground for measurement (not shown in the carton text). As shown in Figure 10b, ρ and H-h constitute the hypotenuse and right angle of a right triangle, respectively. When the hypotenuse of a triangle is constant and the value of the right angle edge of the triangle is changed, the elevation angle will change. Therefore, this experiment is carried out without changing the condition of ρ. Therefore, in this experiment, by changing *h*_2_ to *h*_1_ without changing ρ, different elevation angles can be measured under the condition of constant ρ and θ.

2. Distance Measurement

In order to obtain the different distances between the center of the detector and the target, the detector can be placed on an insulating table or paper box and then drag the insulating table or paper box in the *X* direction to change the distance between the center of the detector and the target. The measurement points are 1.05, 1.5, and 2.25 m at 1.5 times the safety distance, corresponding to 10 kV, 35 kV, and 110 kV, respectively.

3. Azimuth Measurement

The measurement of azimuth is more complicated, because the inverse trigonometric function needs to be solved after solving the tangent value by $\tan\varphi =\frac{\sum_{1}^{n}\frac{\sin\varphi_{i}}{Q_{i}}}{\sum_{1}^{n}\frac{\cos\varphi_{i}}{Q_{i}}}$. According to the nature of the tangent function, it can be seen that there is no unique solution for the target azimuth in the range of 0–360°. Therefore, in the experiment, first determine the number of quadrants in the *XOY* plane of the detector according to the output values of the five sensors, and then use $\tan\varphi =\frac{\sum_{1}^{n}\frac{\sin\varphi_{i}}{Q_{i}}}{\sum_{1}^{n}\frac{\cos\varphi_{i}}{Q_{i}}}$ to solve the azimuth of the target.

The experimental data is shown in Table 5. From the table, the output value of each sensor, the measurement coordinate $P^{\prime }$ of the target, and the measurement error can be obtained. The measurement error is the actual localization parameter of the target minus the absolute value of the measurement localization parameter. It can be seen from Table 5 that under the experimental conditions of this article, when the detector is located at a 10 kV alarm distance, the range error is 0.18 m, the elevation error is 6.8°, and the azimuth error is 4.57°. When the detector is located at the 35 kV warning distance, the distance error is 0.2 m, the elevation error is 4.8°, and the azimuth error is 5.14°.

According to the alarm distance being 1.5 times the safety distance, it is known that the allowable range of the distance error of 10 kV is within 0.35 m, and 35 kV is within 0.5 m. It shows that the localization method can meet at least the requirements of safety distance warning of 10 kV and 35 kV in electric power operation. In Table 5, when the distance from the detector to the ground decreases from *h*_1_ to *h*_2_, the measured value also decreases at the same distance. This is because the field source is closer to the ground and the electric field lines reach the detector as a non-uniform electric field. However, the localization method in this article can still be used. It should be noted that when the azimuth angles are 90° and 270°, no feasible solution can be obtained. In this case, it can only be used for the calculation of distance and elevation angle.

It can also be seen from Table 5 that when the detector is located at the 110 kV alarm distance, the distance error reaches 1.14 m, the elevation error reaches 35.47°, the azimuth error reaches 23.71°, and even the case where the distance and the elevation angle cannot be calculated (Table 5 is displayed as “×”). This is because the experiment uses a 5 kV field source. When the detector is too far away from the field source, the signal received by the sensor is weak, and the slight deviation between the output value of the sensor and the theoretical value will also lead to a large measurement error and even no solution.

It can also be seen from the table that the distance error in the experiment is higher than the simulated distance error, which is due to factors such as temperature, humidity, edge effects, and operating errors.

## 7. Conclusions

(1) Aiming at the AC electric field, a new method for localization of aerial AC targets by the capacitive one-dimensional spherical electric field sensor circular array is studied. By combining the measurement principle of the spherical electric field sensor and the plane circular array theory, a mathematical model for the localization of air targets in an AC electric field is established.

(2) According to the error theory and the research by Chen Xi and others of Beijing Institute of Technology, we know that the main factor that causes errors is the introduction of white noise in the measurement process, and an error model is established through white noise. Starting from the error model, the relationship between the number of sensors, the radius of the array, the radius of the sensor, and the measurement error is analyzed. As the array radius increases, the measurement error gradually decreases.

(3) The objective function and the constraint conditions are determined from the relationship between the mutual interference between the array elements, the minimum induced charge amount, and the distance accuracy. A genetic algorithm was used to optimize the layout parameters of the sensor, and the layout parameters under various constraints were obtained after multiple solutions: the array radius was 0.2 m, the sensor radius was 1.5 cm, and the number of sensors on the circumference was 4. Among them, the distance error is about 8.4%.

(4) A measurement platform was established in the laboratory to verify the localization method of the air AC electric field target. The experimental results show that the distance error is 0.18 m, the elevation error is 6.8°, and the azimuth error is 4.57 ° when the detector is located at a 10 kV alarm distance. At 35 kV alarm distance, the distance error is 0.2 m, the elevation error is 4.8°, and the azimuth error is 5.14°, which meets the safety distance warning requirements of 10 kV and 35 kV voltage levels.

The research process in this article is static. The next step is to study the effects of speed and the detector on different planes during the movement. This method eventually needs to be installed on a worker or a UAV, and can be carried by using a patch-type sensor, but it is necessary to study the influence of the sensor on the surface with different curvature and the error compensation. Next, we should continue to study the error range caused by factors such as temperature, humidity, and edge effects on the surface of the sensor induction board.

## Figures and Tables

**Table 1:** Safety distance when power equipment is not powered off.

**Voltage level (kV)**	**10**	**35**	**110**	**220**
**Safe distance (m)**	0.7	1.0	1.5	3.0

**Table 2:** Optimization model parameter settings.

9	Numerical Value	Parameter	Numerical Value
Q	10^−6^C	$k_{\sigma }$	0.4
$\rho_{0}$	10 m	$k_{R}$	0.3
θ	60°	$k_{r}$	0.2
$\varepsilon_{r}$	$2.5\varepsilon_{0}$	$k_{n}$	0.1
$Q_{\min}$	1.3 × 10^−12^C	r	5 to 30 mm
*φ*	30°	n	4 to 108
$\varepsilon_{0}$	8.58 × 10^−12^	$k_{e}$	0.01
R	0 to 20 cm		

**Table 3:** Genetic algorithm parameter settings.

Parameter	Meaning	Set Value
*m*	Initial population	200
*G*	Maximal genetic algebra	500
$P_{c}$	Crossover probability	80%
$P_{m}$	Mutation probability	1%

**Table 4:** Optimization results.

Serial Number	Distance Accuracy (%)	Array Radius (m)	Sensor Radius (m)	Number of Sensors	Evaluation Function Value
1	7.2	0.2	0.015	4	8.67219
2	10.8	0.199	0.015	4	8.65989
3	11.5	0.199	0.015	4	8.68891
4	8	0.2	0.015	4	8.65807
5	8.4	0.2	0.015	4	8.65588
6	12.4	0.2	0.011	6	11.1286
7	12.2	0.2	0.011	6	11.1308
8	10.6	0.2	0.011	6	11.1282
9	8.5	0.2	0.011	6	11.1253
10	11.5	0.2	0.011	6	11.1303

**Table 5:** Experimental data.

Target Coordinates P	Target Measurement $P^{\prime }$	Sensor0 (kV/m)	Sensor1 (kV/m)	Sensor2 (kV/m)	Sensor3 (kV/m)	Sensor4 (kV/m)	Measurement Error ($\rho^{\prime },\theta^{\prime },\varphi^{\prime }$)
(1.05,35.52,0)	(0.92,30.89,1.16)	3.30	4.01	3.16	2.60	3.13	(0.13,4.63,1.16)
(1.05, 35.52,60)	(1.21,44.12,57.17)	3.30	3.68	3.93	2.88	2.69	(0.16,8.6,4.57)
(1.05,42.55, 0)	(0.87,36.40, 2.29)	2.77	3.58	2.65	2.10	2.64	(0.18,6.15,2.29)
(1.5,24,0)	(1.67,22.33, 5.14)	1.34	1.49	1.30	1.24	1.28	(0.17,1.67,5.14)
(1.5,24,30)	(1.30,18.67,26.10)	1.34	1.4.4	1.36	1.21	1.25	(0.2,4.8,3.9)
(1.5,28.25,0)	(1.31,33.05,−1.72)	1.07	1.25	1.04	0.90	1.05	(0.19,4.8,1.72)
(2.25,15.73,0)	(1.78,51.2,0)	0.45	0.47	0.43	0.42	0.43	(0.47,35.47,0)
(2.2.5,15.73,100)	(×,×,76.29)	0.45	0.46	0.47	0.45	0.43	(×,×,23.71)
(2.25,18.39,0)	(1.11,12.1,−11.86)	0.35	0.37	0.33	0.32	0.33	(1.14,6.29,11.86)
